# SMART coils for intracranial aneurysm repair – a single center experience

**DOI:** 10.1186/s12883-020-1623-9

**Published:** 2020-01-29

**Authors:** Behme Daniel, Sack Henrik, Tsogkas Ioannis, Rohde Veit, Psychogios Marios-Nikos

**Affiliations:** 1 Department of diagnostic and interventional Neuroradiology, University Medical Center Göttingen, Robert Koch Str. 40, 37075 Göttingen, Germany; 2 Department of diagnostic and Interventional Radiology, University Medical Center Göttingen, Robert Koch Str. 40, 37075 Göttingen, Germany; 3 Department of diagnostic and interventional Neuroradiology, University Hospital Basel, Petersgraben 4, 4031 Basel, Basel, Switzerland; 4 Department of Neurosurgery, University Medical Center Göttingen, Robert Koch Str. 40, 37075 Göttingen, Germany

**Keywords:** Intracranial aneurysm, Coil-embolization, New devices, Subarachnoid hemorrhage

## Abstract

**Background:**

Due to uniform stiffness of standard platinum coils, dense packing of intracranial aneurysms can be difficult to achieve, since stiffer coils can cause microcatheter prolapse or coil migration. SMART coils have a varying softness along the length of the coils to improve deliverability. We report our initial 2 year experience with the SMART coil system, including direct and follow-up results.

**Methods:**

We performed a retrospective study of all patients who underwent coil embolization of an intracranial aneurysm with SMART coils between July 2016 and August 2018 at our institution. We analyzed clinical and angiographic data before and directly after treatment as well as at 6 months follow-up.

**Results:**

A total of 49 patients harboring 49 aneurysms were treated; 23 (47%) were ruptured aneurysms. Most aneurysms (57%) were located in the anterior circulation. Median patient age was 55 (31–88), 63% were female. Mean aneurysm size was: neck 3.4 (±1.5), height 6.3 (±2.9) and width 5.2 (±2.3) mm. SMART coils were solely used in 96% of cases. Initial favorable angiographic results were achieved in 45 (92%) of 49 cases, which were stable at 6 months in 26/29 (90%). Thromboembolic complications occurred in 4 (8%) cases without clinical sequelae; microcatheter prolapse occurred in 1 case. No aneurysm rupture or device malfunction was observed.

**Conclusion:**

The treatment of ruptured and unruptured intracranial aneurysms with SMART Coils was safe and efficacious in our cohort.

## Background

Endovascular techniques for intracranial aneurysm (IA) repair have evolved quickly over the past decades. So far only a few of the novel techniques have been investigated in prospective trials [1, 2]. Most novel devices have not been studied in large prospective studies. Nevertheless, utilization, complications and outcomes have to be investigated to determine the safety and efficacy of new devices. A relatively new device to treat IAs is the SMART coil (Penumbra Inc., Alameda, CA, USA). This hybrid coil becomes progressively softer from its distal to its proximal end and should therefore allow easier deployment with a more stable microcatheter positioning compared to standard coils with a uniform stiffness. In addition, SMART coils have a stretch resistant platform, which promises more safety while the coils are placed or removed. SMART coils have been investigated in two case series and promising results were reported regarding safety and angiographic outcomes [3, 4]. However, in both studies the number of cases in which SMART coils were used in conjunction with other coils was rather high with 45% in a series published by Spiotta et al. in 2017 and 18% in the cohort published by Sokolowski et al. this year [3, 4]. In this series 96% were treated solely with SMART coils. Therefore, it might deliver more precise results on safety and direct/long-term angiographic outcome.

## Methods

### Patient selection

We performed a retrospective study of all patients who received coil embolization of an IA with SMART coils between July 2016 and August 2018. Approval of the local ethics committee was obtained (reference number 15/2/19). We treated the first case with SMART coils in mid 2016. After our initial experience, we decided to use it more frequently as in our experience the number of push-backs was lower compared to other coils we used prior. Clinical and demographic information were taken from the electronic patient files and angiographic data including technical details of the intervention were taken from our Picture Archiving and Communication (PACS) system. Patient characteristics included: age, sex, smoking status and co-morbid hypertension. If the aneurysms were ruptured World federation of Neurosurgeons (WFNS) grades were determined. Regarding the aneurysm characteristics the following items were investigated: aneurysm location, aneurysm sizes (neck, height and width), anatomical configuration of the aneurysm (sphere, ellipsoid, bilobulated), presence of a daughter sac and number of additional aneurysms if any were present. Procedural details contained the number of coils, size of coils, the number of SMART coils and use of adjunctive devices such as balloons or stents.

Angiographic Outcome and complications (thromboembolic, aneurysm rupture, microcatheter prolapse) were recorded directly after the procedure and at 6 months follow-up. The rate of loss to follow-up was 40%. Angiographic outcome was measured according to the modified Raymond-Roy classification (MRRC) [5]. MRRC I and II were deemed as favorable outcome.

#### Procedures

All procedures were performed under general anesthesia. In most of the cases (> 90%) a short 6F sheath was introduced into the femoral artery and a 6F Benchmark catheter (Penumbra Inc., Alameda, CA, USA) was advanced to the cervical internal carotid artery (ICA) or vertebral artery (VA) respectively. We used the XT-17 microcatheter (Stryker, Freemont, USA) for aneurysm embolization in most cases (> 90%). In case of a stent assisted coil embolization the Acclino flex Stent (Acandis, Pforzheim, Germany) was used.

#### Data analysis

Descriptive statistics were performed for patient and aneurysm characteristics. Categorical variables were expressed as n/N (%), continuous variables were reported as means ± standard deviation.

## Results

A total of 49 patients harboring 49 aneurysms were treated with SMART Coils between July 2016 and August 2018. Median age was 55 (31–88), 31/49 (63%) were female. Hypertension was present in 25/49 (51%) patients and 18/49 (37%) were current smokers. Aneurysm localizations were as follows: anterior communicating artery (ACOM) in 21/49 (43%), middle cerebral artery (MCA) in 3/49 (6%), internal carotid artery (ICA) in 4/49 (8%), posterior communicating artery (PCOM) in 5/49 (10%), basilar artery (BA) in 9/49 (18%), posterior cerebral artery (PCA) in 4/49 (8%) and posterior inferior cerebellar artery (PICA) in 3/49 (6%) of cases. Eight out of forty nine (16%) patients were harboring at least one additional aneurysm; none of these was treated at the time of treatment with SMART coils. However, the presence of additional aneurysms did not alter our treatment strategy. In case of a ruptured aneurysm and the presence of multiple aneurysms, the ruptured aneurysm was deducted on basis of the bleeding distribution and aneurysm size. Aneurysm shapes were: sphere in 14 (29%), ellipsoid in 25 (51%) and bilobulated in 10 (20%) of cases. Mean aneurysm size was: neck 3.4 (±1.5) mm, height 6.3 (±2.9) mm and width 5.2 (±2.3). A daughter sac was present in 15/49 (31%). Twenty-three of forty-nine patients (47%) presented with acute aneurysm rupture. WFNS grades were: I in 6/23 (26%), II in 7/23 (30%), III in 3/23 (13%), IV in 1/23 (4%) and V in 6/23(26%). (For an overview of baseline characteristics see Table 1).

**Table 1:** Baseline characteristics of patients and aneurysms

Characteristic	n/N (%) or median* (min/max), or mean** (±std. deviation)
Age	55 (31–88)
Female sex	23/49 (63%)
Hypertension	25/49 (51%)
Current smoker	18/49 (37%)
Ruptured aneurysms	23/49 (47%)
WFNS grade I	6/23 (26%)
WFNS grade II	7/23 (30%)
WFNS grade III	3/23 (13%)
WFNS grade IV	1/23 (4%)
WFNS grade V	6/23 (26%)
Aneurysm location	
ACOM	21/49 (43%)
MCA	3/49 (6%)
ICA	4/49 (8%)
PCOM	5/49 (10%)
BA	9/49 (18%)
PCA	4/49 (8%)
PICA	3/49 (6%)

SMART coils only were used in 47/49 (96%) cases. In two cases we used one to two other coils at the end of the procedure. The median number of coils implanted was 5 (2–23).

Balloon assisted coil embolization (BACE) was carried out in 1/49 (2%) and stent assisted coil embolization (SACE) in 21/49 (43%) of cases. Complications were present in 5/49 (10%) patients with 1 microcatheter prolapse and 4 thromboembolic complications of which none had a clinical sequelae. No aneurysm rupture and no technical malfunction of SMART Coils was noted.

Initial angiographic results were: MRRC I in 25/49 (51%), MRRC II in 20/49 (41%), MRRC IIIa in 4/49 (8%). No MRRC IIIb result was observed.

**Fig. 1:**
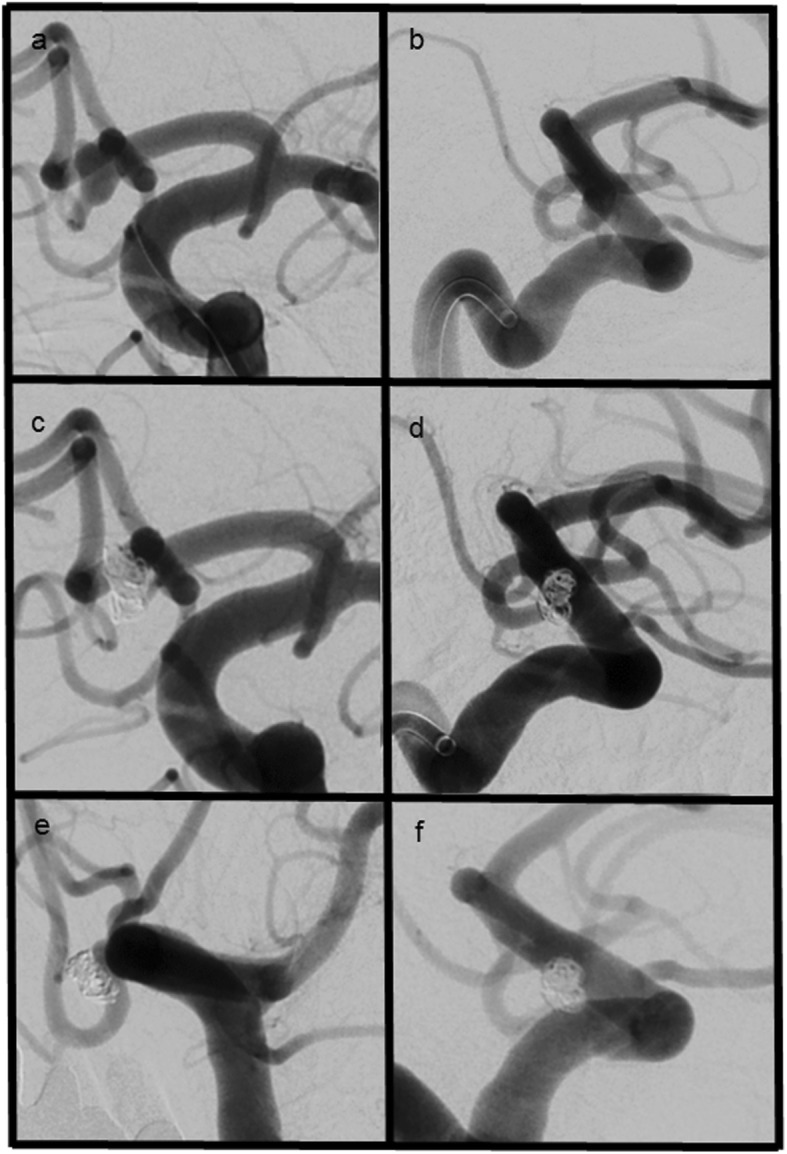
**a** + **b** ap and lateral views of a ruptured ACOM aneurysm (**c** + **d**) ap and lateral views showing complete occlusion of the aneurysm (**e** + **f**) 6 months follow up confirming complete occlusion of the aneurysm

Six month angiographic follow up was available for 29/49 (60%) of patients. In this subgroup initial angiographic results were as follows: 15 cases with MRRC I (52%), 11 with MRRC II (38%), 3 with MRRC IIIa (10%) and 0 with IIIb. After 6 months there were 14 cases with MRRC I (48%), 12 with MRRC II (42%), 2 with MRRC IIIa (7%) and 1 with MRRC IIIb (3%). (An overview of treatment details and results can be found in Table 2, a case example with 6 months follow up is shown in Fig. 1).

**Table 2:** Interventional details and angiographic results

Characteristic	n/N (%) or median* (min/max)
Number of coils implanted	5 (2–23)*
SMART coils only	47/49 (96%)
Complications	
Microcatheter prolapse	1/49 (2%)
Thrombembolic infarcts	4/49 (8%)
Initial angiographic outcome	
MRRC I	25/49 (51%)
MRRC II	20/49 (41%)
MRRC IIIa	3/49 (6%)
MRRC IIIb	0/49 (0%)
Follow up outcome	
MRRC I	14/29 (48%)
MRRC II	12/29 (46%)
MRRC IIIa	2/29 (7%)
MRRC IIIb	1/29 (3%)

### Clinical presentation

Patients with unruptured aneurysms: At admission 16/26 (62%) presented with modified Rankin Scale (mRS) 0, of these all 16 were mRS 0 post-operatively. Within this group 6 month follow up was available for 12/16. 1 patient presented with mRS 1 and the remaining 11 still with mRS 0. 3/26 (12%) presented with mRS 1 at admission and post-operatively. Follow up of these was only available for 1 patient who was presenting with mRS 0 at 6 months. 5/26 (19%) presented with mRS 2, of which 4 remained at mRS 2 post-operatively and 2 patients improved to mRS2, which did not change at 6 months follow up. One patients presented with mRS 3 pre- and post-operatively and another with mRS 4. Follow up was only available for the patient with an initial mRS of 3 which decreased to 4 at 6 months follow up.

For those patients with ruptured aneurysms: pre-operative: no patient with mRS 0, 6/23 (26%) with mRS1 and 6/23 (26%) with mRS2. mRS 3 in 3/26 (12%) and mRS 5 in 8/26 (31%). Post-operative (discharge) mRS were 0 in 7/26 (27%), 1 in 4/26 (15%), 2 in 3/26 (12%) and 6 in 6/26 (23%). In this cohort 6 months follow up was available for 10 patients who presented with mRS 0 in 7/10 (70%), mRS 1 in 2/10 (20%) and mRS 2 in 1/10 (10%).

### Discussion

Endovascular treatment of ruptured aneurysms has become an accepted alternative to microsurgical clipping after the publication of the International Subarachnoid Aneurysm Trial (ISAT) almost 30 years ago [6]. However, whether and which unruptured IAs should be treated is still unclear. Several factors contribute to the rupture risk of IAs, including: smoking, sex, age, ethnicity, co-morbidities, family history and others [7]. In addition there is no clear evidence if microsurgical clipping or endovascular treatment is the better option for the treatment of unruptured IAs [8]. Although endovascular treatment methods changed rapidly over the last decade with several new devices that have been introduced into the market [9–11], coil embolization still remains the standard technique for IA embolization. Since the first generation of coils several innovative coils like bioactive coils have been introduced into clinical practice. So far without significant benefits regarding angiographic outcomes [12]. Although occlusion rates of up to 90% (MRRC I + II) have been reported, if standard coil embolization has been carried out, some technical challenges remain [13]. Beside aneurysm rupture, microcatheter prolapse during coil embolization is one important complication. It might lead to other complications like intimal scathing with resulting thrombosis or the need for a repeated navigation into the aneurysm sac, which can be impossible sometimes. To improve coil delivery and to avoid microcatheter prolapse Penumbra developed the SMART Coil, which gets softer from distal to proximal (approximately with a gradient of 3:1). This is the second study investigating SMART coils, which includes long-term follow up. At 6 months we found a rate of 26/29 (90%) MRRC I or II results. Compared to the initial 92% MRRC I + II results in our study, we were able to show that most initially favorable results remained stable. These findings confirm recent results from Sokolowski et al., who reported a rate of 88% favorable long-term angiographic results after embolization with SMART Coils [3]. However, around 10% of patients have insufficient occlusion results and have to be retreated. This is an acceptable rate compared to the literature [14]. Different to the study of Sokolowski et al. we treated most patients with SMART coils only (96% vs 80%), which might had an impact on the results and could explain the slightly better occlusion results we observed [3]. In another study Stapleton et al. found 100% of the “coil only” group to have an initially favorable angiographic outcome [5]. This shows that simple angiographic outcome measurements have to be interpreted cautious and adjunctive devices and aneurysm configuration should always be considered.

There was no device specific complication in our cohort. However, one microcatheter prolapse occurred which was without clinical sequelae. Potentially microcatheter prolapse may lead to relevant complications and so a low rate of re-navigation into the aneurysm should be desirable. Our study thereby confirms what has been reported by others [3]. There was no coil stretching in our cohort as well, which may indicate a favorable behavior of SMART coils during withdrawal into to the microcatheter. Regarding the thromboembolic complications too many confounding factors exist to correlate those events to a specific coil. Nevertheless, the rate of 4/49 (8%) is comparable to what has been found in other cohort studies with SMART coils [3].

Our study has several limitations. First selection bias cannot be ruled out due to the retrospective single center design, the small cohort and the high rate of loss to follow-up of up (40%). Second the results and the complications were reported by the operators and treating physicians. No core-lab evaluation was performed. In addition, there was a learning curve over the time we treated patients with SMART coils, which might have influenced our results.

A prospective multi-center study in the U.S.A. is already initiated and hopefully the results of this study will allow further insight into the advantages/disadvantages of SMART coils.

## Conclusion

In our cohort the treatment of ruptured and unruptured IAs with SMART Coils was safe and led to a high rate of initial and follow-up occlusions.

## Data Availability

The datasets used and/or analysed during the current study are available from the corresponding author on reasonable request.

## Abbreviations

BA: Basilar artery
BACE: Balloon assisted coil embolization
IA: Intracranial aneurysm
ICA: Internal carotid artery
ISAT: International Subarachnoid Aneurysm Trial
MCA: Middle cerebral artery
MRRC: Modified Raymond-Roy classification
PACS: Picture archiving and communication system
PCA: Posterior cerebral artery
PCOM: Posterior communicating artery
PICA: Posterior inferior cerebellar artery
SACE: Stent assisted coil embolization
VA: Vertebral artery
WFNS: Word federation of Neurosurgeons
