# Correction to: SMART coils for intracranial aneurysm repair - a single center experience

**DOI:** 10.1186/s12883-020-01767-4

**Published:** 2020-05-14

**Authors:** Daniel Behme, Henrik Sack, Ioannis Tsogkas, Veit Rohde, Marios-Nikos Psychogios

**Affiliations:** 1 Department of Diagnostic and Interventional Neuroradiology, University Medical Center Göttingen, Robert-Koch-Str. 40, 37075 Göttingen, Germany; 2 Department of Diagnostic and Interventional Radiology, University Medical Center Göttingen, Robert-Koch-Str. 40, 37075 Göttingen, Germany; 3 Department of Diagnostic and Interventional Neuroradiology, University Hospital Basel, Petersgraben 4, 4031 Basel, Switzerland; 4 Department of Neurosurgery, University Medical Center Göttingen, Rober-Koch-Str. 40, 37075 Göttingen, Germany

**Correction to: BMC Neurol 20, 38 (2020)**

**https://doi.org/10.1186/s12883-020-1623-9**

Following publication of the original article [1], the authors opted to correct the interchanged given names and family names.
